# Supplementary material for: Quantifying within-host diversity of H5N1 influenza viruses in humans and poultry in Cambodia
Source: PLoS Pathog. 2020 Jan 17;16(1):e1008191. doi: 10.1371/journal.ppat.1008191 (PMC6992230; doi:10.1371/journal.ppat.1008191)
Supplement: S1 Fig — The mean coverage depth at each nucleotide site (x-axis) for each gene across our 8 human and 5 duck samples is shown. Solid black lines represent the mean coverage across samples, and the grey shaded area represents the standard deviation of coverage depth across samples. (PDF) [file ppat.1008191.s001.pdf]

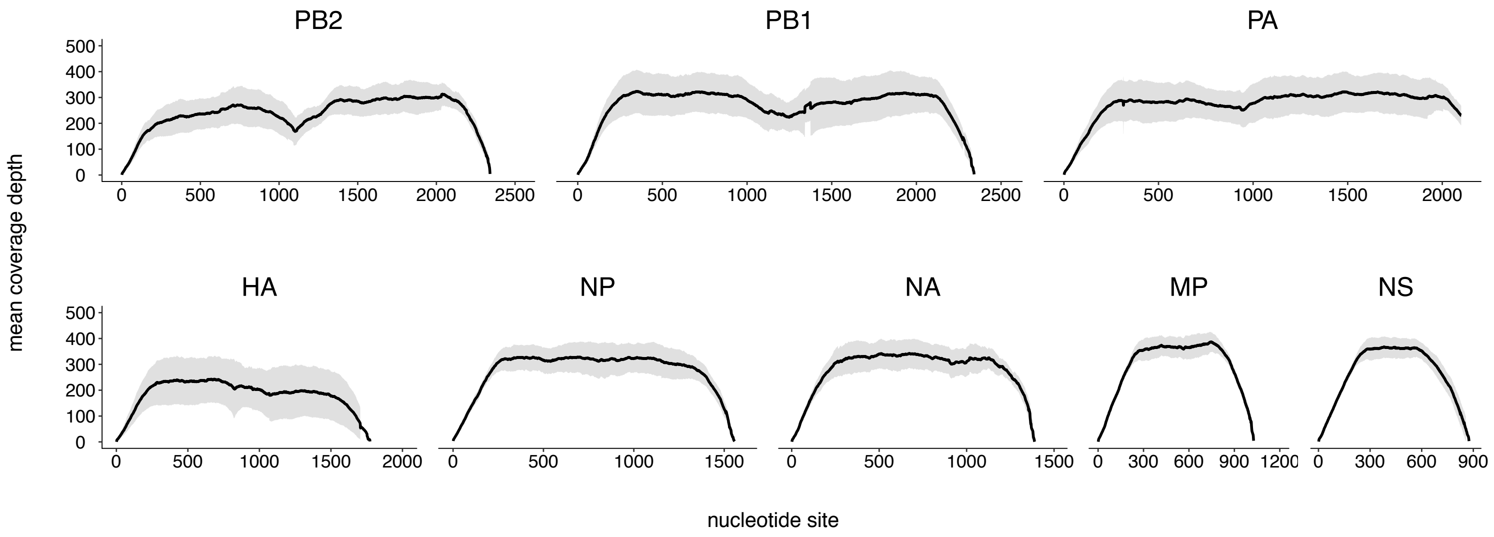

Figure S1: Genome coverage

The mean coverage depth at each nucleotide site (x-axis) for each gene across our 8 human and 5 duck samples is shown. Solid black lines represent the mean coverage across samples, and the grey shaded area represents the standard deviation of coverage depth across samples.
