# Supplementary material for: Quantifying within-host diversity of H5N1 influenza viruses in humans and poultry in Cambodia
Source: PLoS Pathog. 2020 Jan 17;16(1):e1008191. doi: 10.1371/journal.ppat.1008191 (PMC6992230; doi:10.1371/journal.ppat.1008191)
Supplement: S2 Fig — All currently available H5N1 virus sequences were downloaded from the Influenza Research Database and the Global Initiative on Sharing All Influenza Data and used to generate full genome phylogenies using Nextstrain’s augur pipeline. Colors represent the geographic region in which the sample was collected (for tips) or the inferred geographic location (for internal nodes). The x-axis position indicates the date of sample collection (for tips) or the inferred time to the most recent common ancestor (for internal nodes). In the full phylogenies (left), H5N1 viruses from Cambodia selected for within-host analysis are indicated by tan circles with black outlines. The subtrees containing the Cambodian samples selected for within-host analysis are shown to the right in the order that they appear in the full tree. In these trees, human tips are marked with a tan circle with a black outline, while duck tips are denoted with a tan square with a black outline. Both human and duck tips are labelled with their strain names. Internal genes from samples collected prior to 2013 belong to clade 1.1.2, while internal genes from samples collected in 2013 or later belong to clade 2.3.2.1a. All HA and NA sequences in this dataset, besides A/duck/Cambodia/Y0224304/2014, belong to clade 1.1.2. (PDF) [file ppat.1008191.s002.pdf]

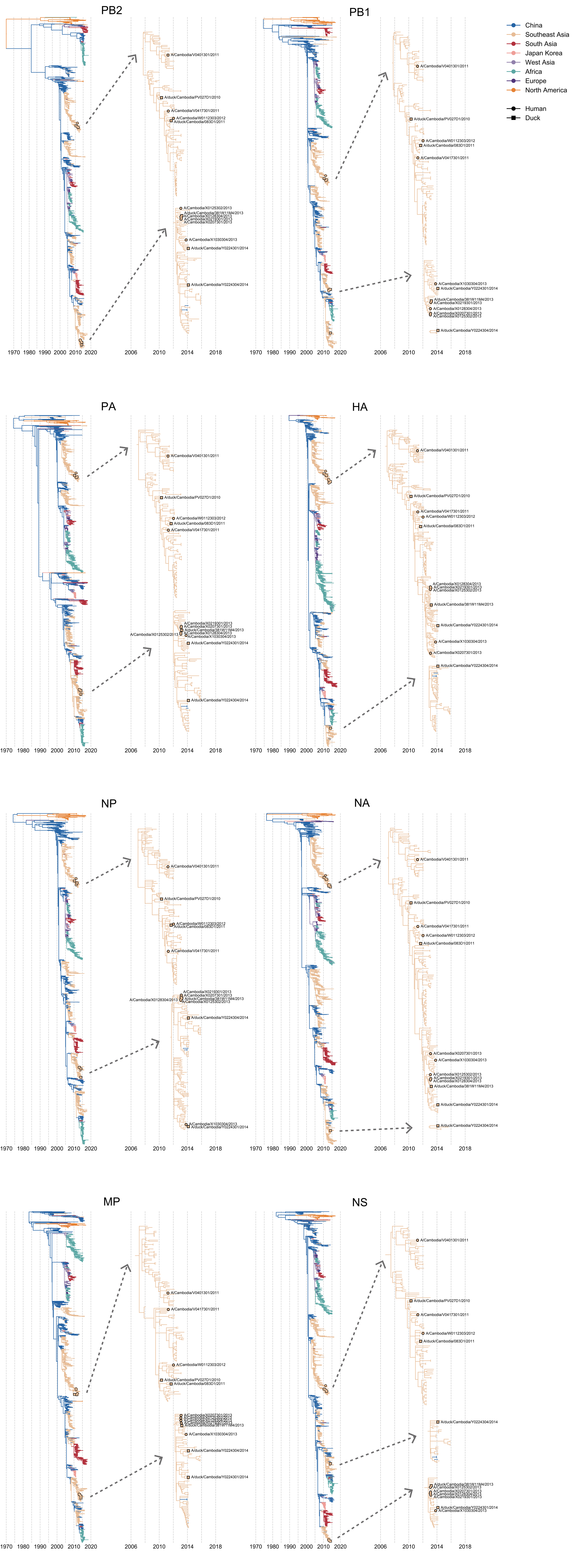

Figure S2: Full genome phylogenetic placement of H5N1 virus samples from Cambodia
All currently available H5N1 virus sequences were downloaded from the Influenza Research Database and the Global Initiative on Sharing All Influenza Data and used to generate full genome phylogenies using Nextstrain's augur pipeline. Colors represent the geographic region in which the sample was collected (for tips) or the inferred geographic location (for internal nodes). The x-axis position indicates the date of sample collection (for tips) or the inferred time to the most recent common ancestor (for internal nodes). In the full phylogenies (left), H5N1 viruses from Cambodia selected for within-host analysis are indicated by tan circles with black outlines. The subtrees containing the Cambodian samples selected for within-host analysis are shown to the right in the order that they appear in the full tree. In these trees, human tips are marked with a red circle, while duck tips are denoted with a blue circle. Internal genes from samples collected prior to 2013 belong to clade 1.1.2, while internal genes from samples collected in 2013 or later belong to clade 2.3.2.1a. All HA and NA sequences in this dataset, besides A/duck/Cambodia/Y0224304/2014, belong to clade 1.1.2.
