# Supplementary material for: Quantifying within-host diversity of H5N1 influenza viruses in humans and poultry in Cambodia
Source: PLoS Pathog. 2020 Jan 17;16(1):e1008191. doi: 10.1371/journal.ppat.1008191 (PMC6992230; doi:10.1371/journal.ppat.1008191)
Supplement: S3 Fig — All within-host variants detected in our study are shown. Each row represents one sample and each column represents one gene. The x-axis shows the nucleotide site and the y-axis shows the frequency that the variant was detected within-host. Filled circles represent nonsynonymous changes, while open circles represent synonymous changes. Blue dots represent variants identified within duck samples, while red dots represent variants identified in human samples. Blank plots indicate that no variants were identified in that sample and gene. (PDF) [file ppat.1008191.s003.pdf]

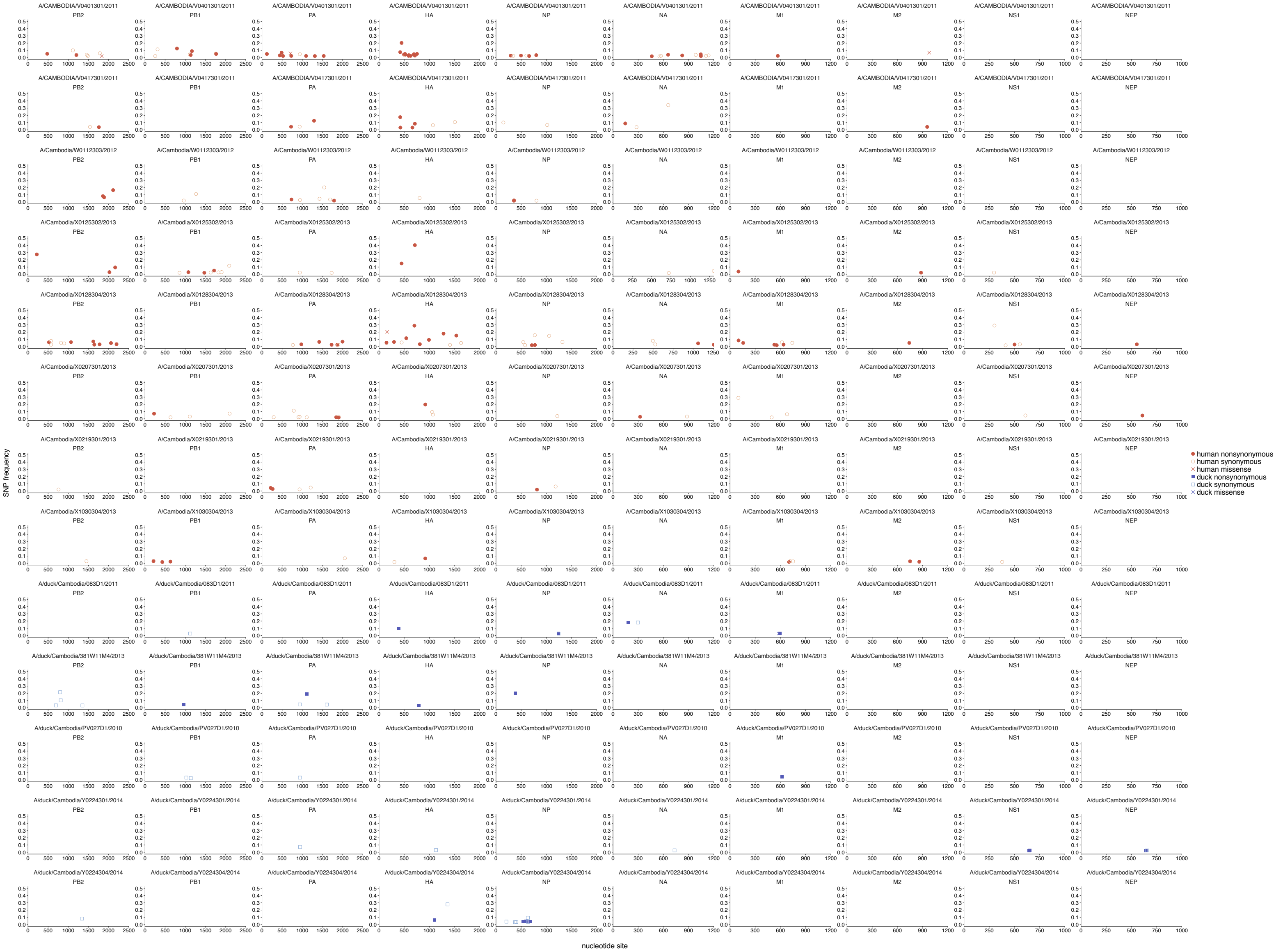

Figure S3: All within-host variants detected in our dataset

All within-host variants detected in our study are shown. Each row represents one sample and each column represents one gene. The x-axis shows the nucleotide site and the y-axis shows the frequency that the variant was detected within-host. Filled circles represent nonsynonymous changes, while open circles represent synonymous changes. Blue dots represent variants identified within duck samples, while red dots represent variants identified in human samples. Blank plots indicate that no variants were identified in that sample and gene.
