# Supplementary material for: Quantifying within-host diversity of H5N1 influenza viruses in humans and poultry in Cambodia
Source: PLoS Pathog. 2020 Jan 17;16(1):e1008191. doi: 10.1371/journal.ppat.1008191 (PMC6992230; doi:10.1371/journal.ppat.1008191)
Supplement: S4 Fig — (a) For each human sample, the full genome nucleotide diversity (πΝ or πS) is plotted vs. the days post-symptom onset. Dark red dots represent the mean, full-genome nonsynonymous diversity for a given sample (πΝ), and light red dots represent the mean, full-genome synonymous diversity for that same sample (πS). Neither nonsynonymous nor synonymous diversity are correlated with days post symptom onset (nonsynonymous: r2 = -0.17, p = 0.69; synonymous: r2 = -0.22, p = -0.61). (b) To compare whether the number of putative host-adapting mutations increased over time in humans, we compared the number of host-specific and non-host specific mutations in humans sampled either in “early infection” (5–8 days post symptom onset), or in “late infection” (9–12 days post symptom onset). We divided the data into these categories by splitting on the mean days post symptom onset for human samples, which was 8 days. We then compared the proportion of host-specific variants during early and late infections with a Fisher’s exact test. The proportion of variants that are host-specific is not different in early vs. late infections (p = 0.72). (PDF) [file ppat.1008191.s004.pdf]

a

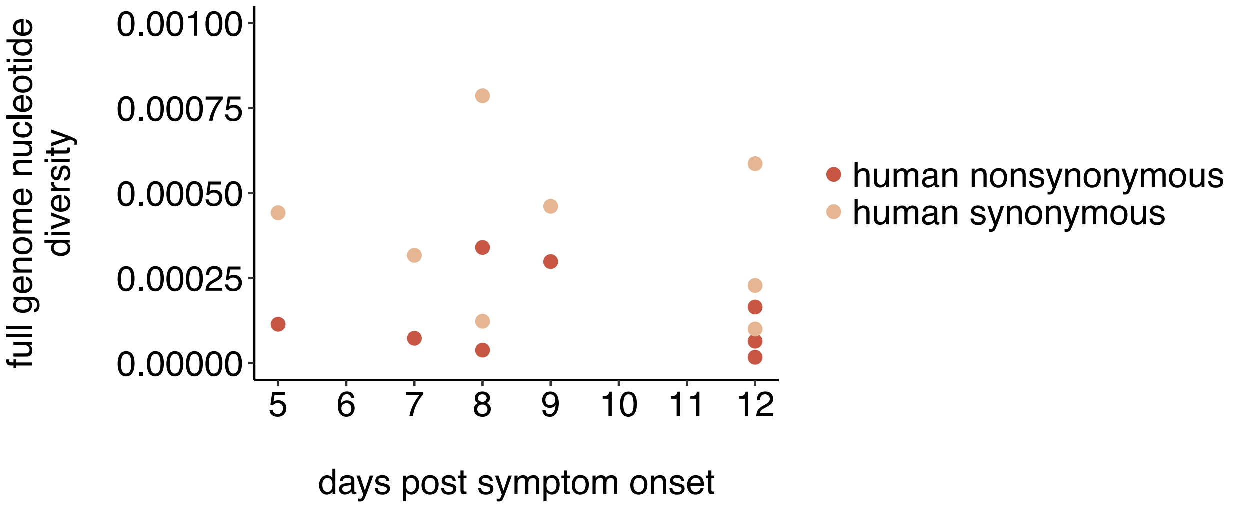

b

	"early infection" (5-8 days)	"late infection" (9-12 days)
host-specific mutations	18	22
non-host specific mutations	82	84

Figure S4: Neither diversity nor host-specific mutations increase over time
(a) For each human sample, the full genome nucleotide diversity (π_N or π_S) is plotted vs. the days post-symptom onset. Dark red dots represent the mean, full-genome nonsynonymous diversity for a given sample (π_N), and light red dots represent the mean, full-genome synonymous diversity for that same sample (π_S). Neither nonsynonymous nor synonymous diversity are correlated with days post symptom onset (nonsynonymous: r^2 = -0.17, p = 0.69; synonymous: r^2 = -0.22, p = -0.61). **(b)** To compare whether the number of putative host-adapting mutations increased over time in humans, we compared the number of host-specific and non-host specific mutations in humans sampled either in "early infection" (5-8 days post symptom onset), or in "late infection" (9-12 days post symptom onset). We divided the data into these categories by splitting on the mean days post symptom onset for human samples, which was 8 days. We then compared the proportion of host-specific variants during early and late infections with a Fisher's exact test. The proportion of variants that are host-specific is not different in early vs. late infections (p = 0.72).
