# Supplementary material for: Quantifying within-host diversity of H5N1 influenza viruses in humans and poultry in Cambodia
Source: PLoS Pathog. 2020 Jan 17;16(1):e1008191. doi: 10.1371/journal.ppat.1008191 (PMC6992230; doi:10.1371/journal.ppat.1008191)
Supplement: S1 Table — Every SNV identified in humans and ducks within-host are displayed with their frequency, coding region change, and functional annotation. All annotations for H5 HAs, N1 NAs, and all subtypes for all other genes were downloaded from the Influenza Research Database Sequence Feature Variant Types tool. Each SNV was then annotated as shown in the “description” column. These descriptions are paraphrased from annotations presented in the Influenza Research Database. We then manually curated annotated mutations to determine whether they were involved in “host-specific” functions or not, as shown in the “host-specific?” column. We defined host-specific functions/interactions as receptor binding, interaction with host cellular machinery, nuclear import and export, immune antagonism, 5’ cap binding, temperature sensitivity, and glycosylation. We also included sites that have been phenotypically identified as determinants of transmissibility and virulence. Sites that participate in binding interactions with other viral subunits or vRNP, conserved active site domains, drug resistance mutations, and epitope sites were not categorized as host-specific for this analysis. We annotated both synonymous and nonsynonymous mutations in our dataset. (DOCX) [file ppat.1008191.s005.docx]

**Table S1: All within-host SNVs with annotations**

| sample name | gene | site | ref | var | aa change | change type | freq (%) | description | Host-specific? |
| --- | --- | --- | --- | --- | --- | --- | --- | --- | --- |
| A/Cambodia/X0125302/2013 | PB2 | 220 | T | C | Met66Thr | nonsynonymous | 27.59% | No known function | no |
| A/Cambodia/V0401301/2011 | PB2 | 478 | A | C | His151Pro | nonsynonymous | 5.26% | No known function | no |
| A/Cambodia/X0128304/2013 | PB2 | 521 | A | T | Glu165Val | nonsynonymous | 5.88% | No known function | no |
| A/Cambodia/X0128304/2013 | PB2 | 573 | A | G | Gln182Gln | synonymous | 7.79% | No known function | no |
| A/Cambodia/X0128304/2013 | PB2 | 574 | T | C | Leu183Leu | synonymous | 2.61% | No known function | no |
| A/duck/Cambodia/381W11M4/2013 | PB2 | 693 | C | T | Gly222Gly | synonymous | 3.27% | No known function | no |
| A/Cambodia/X0219301/2013 | PB2 | 758 | T | G | Thr245Thr | synonymous | 2.18% | No known function | no |
| A/duck/Cambodia/381W11M4/2013 | PB2 | 798 | A | G | Gln257Gln | synonymous | 21.50% | No known function | no |
| A/duck/Cambodia/381W11M4/2013 | PB2 | 813 | T | C | Ala262Ala | synonymous | 10.28% | No known function | no |
| A/Cambodia/X0128304/2013 | PB2 | 819 | A | G | Arg264Arg | synonymous | 5.31% | No known function | no |
| A/Cambodia/X0128304/2013 | PB2 | 897 | T | C | Gly290Gly | synonymous | 4.39% | No known function | no |
| A/Cambodia/X0128304/2013 | PB2 | 1069 | A | T | Asn348Tyr | nonsynonymous | 6.15% | Putative m7GTP cap binding site. | yes |
| A/Cambodia/V0401301/2011 | PB2 | 1115 | C | T | Phe363Phe | synonymous | 10% | Putative m7GTP cap binding site. | yes |
| A/Cambodia/V0401301/2011 | PB2 | 1202 | A | C | Gln392His | nonsynonymous | 3.61% | Putative m7GTP cap binding site. | yes |
| A/duck/Cambodia/Y0224304/2014 | PB2 | 1340 | C | T | Asp441Asp | synonymous | 8.16% | Putative m7GTP cap binding site. | yes |
| A/duck/Cambodia/381W11M4/2013 | PB2 | 1350 | C | T | Asp441Asp | synonymous | 3.19% | Putative m7GTP cap binding site. | yes |
| A/Cambodia/X1030304/2013 | PB2 | 1451 | A | G | Arg479Arg | synonymous | 2.87% | Nuclear localization motif. | yes |
| A/Cambodia/V0401301/2011 | PB2 | 1460 | A | G | Val478Val | synonymous | 3.90% | Nuclear localization motif. | yes |
| A/Cambodia/V0401301/2011 | PB2 | 1484 | T | C | Asp486Asp | synonymous | 2.30% | Nuclear localization motif. | yes |
| A/Cambodia/V0417301/2011 | PB2 | 1539 | C | A | Arg505Arg | synonymous | 4.05% | No known function | no |
| A/Cambodia/X0128304/2013 | PB2 | 1621 | T | C | Ser532Pro | nonsynonymous | 6.90% | No known function | no |
| A/Cambodia/X0128304/2013 | PB2 | 1646 | A | G | Asn540Ser | nonsynonymous | 2.65% | No known function | no |
| A/Cambodia/V0417301/2011 | PB2 | 1761 | C | T | Pro579Ser | nonsynonymous | 3.80% | No known function | no |
| A/Cambodia/X0128304/2013 | PB2 | 1778 | T | C | Val584Ala | nonsynonymous | 3.01% | No known function | no |
| A/Cambodia/V0401301/2011 | PB2 | 1784 | A | G | Lys586Lys | synonymous | 6.01% | No known function | no |
| A/Cambodia/V0401301/2011 | PB2 | 1830 | C | T | Gln602Stop | stop gained | 2.09% | No known function | no |
| A/Cambodia/W0112303/2012 | PB2 | 1859 | T | C | Ile616Thr | nonsynonymous | 8.43% | No known function | no |
| A/Cambodia/W0112303/2012 | PB2 | 1891 | G | A | Glu627Lys | nonsynonymous | 6.63% | A Lys at 627 enhances mammalian replication. | yes |
| A/Cambodia/X0125302/2013 | PB2 | 2022 | G | A | Val667Ile | nonsynonymous | 2.99% | An Ile at 667 was associated with human-infecting H5N1 strain. | yes |
| A/Cambodia/X0128304/2013 | PB2 | 2060 | A | T | Asp678Val | nonsynonymous | 4.80% | No known function | no |
| A/Cambodia/W0112303/2012 | PB2 | 2113 | A | G | Asn701Asp | nonsynonymous | 16.49% | An Asn at 701 enhances mammalian replication. | yes |
| A/Cambodia/X0125302/2013 | PB2 | 2163 | A | G | Ser714Gly | nonsynonymous | 9.59% | An Arg at 714 enhances mammalian replication. | yes |
| A/Cambodia/X0128304/2013 | PB2 | 2198 | T | A | Val724Glu | nonsynonymous | 3.41% | No known function | no |
| A/Cambodia/X1030304/2013 | PB1 | 212 | G | A | Gly71Glu | nonsynonymous | 3.12% | No known function | no |
| A/Cambodia/X0207301/2013 | PB1 | 226 | G | A | Asp76Asn | nonsynonymous | 7.14% | No known function | no |
| A/Cambodia/V0401301/2011 | PB1 | 255 | A | G | Thr85Thr | synonymous | 2.12% | No known function | no |
| A/Cambodia/V0401301/2011 | PB1 | 312 | A | G | Glu104Glu | synonymous | 11.42% | No known function | no |
| A/Cambodia/X1030304/2013 | PB1 | 431 | C | T | Ala144Val | nonsynonymous | 1.78% | No known function | no |
| A/Cambodia/X1030304/2013 | PB1 | 631 | A | G | Arg211Gly | nonsynonymous | 2.34% | Nuclear localization motif. | yes |
| A/Cambodia/X0207301/2013 | PB1 | 634 | C | T | Leu212Leu | synonymous | 2.10% | Nuclear localization motif. | yes |
| A/Cambodia/V0401301/2011 | PB1 | 794 | A | G | Lys265Arg | nonsynonymous | 12.65% | No known function | no |
| A/Cambodia/X0125302/2013 | PB1 | 857 | A | G | Lys279Lys | synonymous | 2.14% | No known function | no |
| A/Cambodia/W0112303/2012 | PB1 | 963 | G | A | Gln313Gln | synonymous | 2.10% | No known function | no |
| A/duck/Cambodia/381W11M4/2013 | PB1 | 968 | A | G | Met317Val | nonsynonymous | 4.21% | No known function | no |
| A/duck/Cambodia/PV027D1/2010 | PB1 | 1026 | G | A | Arg334Arg | synonymous | 3.30% | No known function | no |
| A/Cambodia/X0125302/2013 | PB1 | 1078 | A | G | Lys353Arg | nonsynonymous | 2.94% | An Arg at 353 is associated with higher replication and pathogenicity of an H1N1 pandemic strain. | yes |
| A/Cambodia/V0401301/2011 | PB1 | 1113 | A | G | Glu371Glu | synonymous | 4.33% | No known function | no |
| A/Cambodia/X0207301/2013 | PB1 | 1113 | A | G | Glu371Glu | synonymous | 3.15% | No known function | no |
| A/duck/Cambodia/083D1/2011 | PB1 | 1121 | A | G | Glu371Glu | synonymous | 2.71% | No known function | no |
| A/Cambodia/V0401301/2011 | PB1 | 1136 | A | G | Lys379Arg | nonsynonymous | 3.64% | No known function | no |
| A/duck/Cambodia/PV027D1/2010 | PB1 | 1137 | A | G | Glu371Glu | synonymous | 2.59% | No known function | no |
| A/Cambodia/V0401301/2011 | PB1 | 1165 | A | G | Ile389Val | nonsynonymous | 8.87% | No known function | no |
| A/Cambodia/W0112303/2012 | PB1 | 1267 | C | T | Leu415Leu | synonymous | 11.21% | No known function | no |
| A/Cambodia/X0125302/2013 | PB1 | 1472 | A | G | Ile484Met | nonsynonymous | 2.17% | No known function | no |
| A/Cambodia/X0125302/2013 | PB1 | 1631 | C | T | Asn537Asn | synonymous | 2.50% | No known function | no |
| A/Cambodia/X0125302/2013 | PB1 | 1716 | A | T | Thr566Ser | nonsynonymous | 5.20% | An Ala at 566 is associated with higher replication and pathogenicity of an H1N1 pandemic virulence. | yes |
| A/Cambodia/V0401301/2011 | PB1 | 1758 | A | G | Lys586Lys | synonymous | 5.70% | No known function | no |
| A/Cambodia/V0401301/2011 | PB1 | 1766 | T | C | Leu589Pro | nonsynonymous | 5% | No known function | no |
| A/Cambodia/X0125302/2013 | PB1 | 1823 | C | T | Ile601Ile | synonymous | 2.75% | No known function | no |
| A/Cambodia/X0125302/2013 | PB1 | 1901 | T | C | Pro627Pro | synonymous | 3.08% | No known function | no |
| A/Cambodia/X0125302/2013 | PB1 | 2090 | A | G | Gln690Gln | synonymous | 11.89% | 690 falls into a region of PB1 that binds to PB2 and is critical for PB2 enzymatic activity. | no |
| A/Cambodia/X0207301/2013 | PB1 | 2100 | T | C | Phe700Phe | synonymous | 7.20% | 700 falls into a region of PB1 that binds to PB2 and is critical for PB2 enzymatic activity. | no |
| A/Cambodia/V0401301/2011 | PA | 122 | T | C | Phe35Ser | nonsynonymous | 5% | No known function | no |
| A/Cambodia/X0219301/2013 | PA | 215 | C | T | Pro68Leu | nonsynonymous | 4.32% | No known function | no |
| A/Cambodia/X0219301/2013 | PA | 265 | A | G | Thr85Ala | nonsynonymous | 2.84% | An Ile at 85 enhances polymerase activity of pandemic H1N1 in mammalian cell. | yes |
| A/Cambodia/X0207301/2013 | PA | 291 | T | C | Ser93Ser | synonymous | 2.22% | No known function | no |
| A/Cambodia/V0401301/2011 | PA | 443 | G | A | Arg142Lys | nonsynonymous | 3.07% | No known function | no |
| A/Cambodia/V0401301/2011 | PA | 488 | C | A | Thr157Asn | nonsynonymous | 6.85% | No known function | no |
| A/Cambodia/V0401301/2011 | PA | 523 | G | A | Ala169Thr | nonsynonymous | 2.24% | No known function | no |
| A/Cambodia/V0401301/2011 | PA | 706 | A | T | Arg230Stop | stop gained | 5.94% | No known function | no |
| A/Cambodia/V0417301/2011 | PA | 723 | A | G | Lys237Glu | nonsynonymous | 4.36% | No known function | no |
| A/Cambodia/V0401301/2011 | PA | 727 | A | G | Lys237Glu | nonsynonymous | 2.34% | No known function | no |
| A/Cambodia/W0112303/2012 | PA | 732 | A | G | Lys237Glu | nonsynonymous | 3.64% | No known function | no |
| A/Cambodia/X0128304/2013 | PA | 765 | T | C | Ser247Ser | synonymous | 2.24% | No known function | no |
| A/Cambodia/X0207301/2013 | PA | 789 | A | G | Pro259Pro | synonymous | 11.21% | This site falls into a region of PA that interacts with PB1 residues 1-25, and this interaction is critical for transcription. | no |
| A/Cambodia/X0207301/2013 | PA | 906 | G | A | Glu298Glu | synonymous | 2.26% | This site falls into a region of PA that interacts with PB1 residues 1-25, and this interaction is critical for transcription. | no |
| A/Cambodia/X0207301/2013 | PA | 933 | A | G | Ala307Ala | synonymous | 2.80% | This site falls into a region of PA that interacts with PB1 residues 1-25, and this interaction is critical for transcription. | no |
| A/Cambodia/X0219301/2013 | PA | 933 | A | G | Ala307Ala | synonymous | 2.37% | This site falls into a region of PA that interacts with PB1 residues 1-25, and this interaction is critical for transcription. | no |
| A/Cambodia/V0417301/2011 | PA | 935 | A | G | Ala307Ala | synonymous | 4.38% | This site falls into a region of PA that interacts with PB1 residues 1-25, and this interaction is critical for transcription. | no |
| A/Cambodia/V0401301/2011 | PA | 939 | A | G | Ala307Ala | synonymous | 4.67% | This site falls into a region of PA that interacts with PB1 residues 1-25, and this interaction is critical for transcription. | no |
| A/Cambodia/X0125302/2013 | PA | 939 | A | G | Ala307Ala | synonymous | 2.50% | This site falls into a region of PA that interacts with PB1 residues 1-25, and this interaction is critical for transcription. | no |
| A/duck/Cambodia/381W11M4/2013 | PA | 939 | A | G | Ala307Ala | synonymous | 4.55% | This site falls into a region of PA that interacts with PB1 residues 1-25, and this interaction is critical for transcription. | no |
| A/duck/Cambodia/PV027D1/2010 | PA | 941 | A | G | Ala307Ala | synonymous | 3.31% | This site falls into a region of PA that interacts with PB1 residues 1-25, and this interaction is critical for transcription. | no |
| A/Cambodia/W0112303/2012 | PA | 944 | A | G | Ala307Ala | synonymous | 2.85% | This site falls into a region of PA that interacts with PB1 residues 1-25, and this interaction is critical for transcription. | no |
| A/duck/Cambodia/Y0224301/2014 | PA | 945 | A | G | Ala307Ala | synonymous | 7.28% | This site falls into a region of PA that interacts with PB1 residues 1-25, and this interaction is critical for transcription. | no |
| A/Cambodia/X0128304/2013 | PA | 976 | A | G | Lys318Glu | nonsynonymous | 3.05% | This site falls into a region of PA that interacts with PB1 residues 1-25, and this interaction is critical for transcription. | no |
| A/Cambodia/V0401301/2011 | PA | 1094 | A | C | Asn359Thr | nonsynonymous | 2.26% | This site falls into a region of PA that interacts with PB1 residues 1-25, and this interaction is critical for transcription. | no |
| A/Cambodia/X0207301/2013 | PA | 1110 | G | A | Leu366Leu | synonymous | 2.08% | This site falls into a region of PA that interacts with PB1 residues 1-25, and this interaction is critical for transcription. | no |
| A/duck/Cambodia/381W11M4/2013 | PA | 1118 | G | A | Arg367Lys | nonsynonymous | 19% | This site falls into a region of PA that interacts with PB1 residues 1-25, and this interaction is critical for transcription. | no |
| A/Cambodia/X0219301/2013 | PA | 1209 | G | A | Lys399Lys | synonymous | 4.79% | This site falls into a region of PA that interacts with PB1 residues 1-25, and this interaction is critical for transcription. | no |
| A/Cambodia/V0417301/2011 | PA | 1292 | T | A | Asp426Glu | nonsynonymous | 12.60% | This site falls into a region of PA that interacts with PB1 residues 1-25, and this interaction is critical for transcription. | no |
| A/Cambodia/V0401301/2011 | PA | 1312 | G | A | Val432Ile | nonsynonymous | 2.08% | This site falls into a region of PA that interacts with PB1 residues 1-25, and this interaction is critical for transcription. | no |
| A/Cambodia/X0128304/2013 | PA | 1420 | A | C | Asn466His | nonsynonymous | 6.45% | This site falls into a region of PA that interacts with PB1 residues 1-25, and this interaction is critical for transcription. | no |
| A/Cambodia/W0112303/2012 | PA | 1428 | C | T | Leu469Leu | synonymous | 4.59% | This site falls into a region of PA that interacts with PB1 residues 1-25, and this interaction is critical for transcription. | no |
| A/Cambodia/V0401301/2011 | PA | 1533 | A | G | Ile505Met | nonsynonymous | 2.24% | This site falls into a region of PA that interacts with PB1 residues 1-25, and this interaction is critical for transcription. | no |
| A/Cambodia/W0112303/2012 | PA | 1544 | A | G | Gly507Gly | synonymous | 20.26% | This site falls into a region of PA that interacts with PB1 residues 1-25, and this interaction is critical for transcription. | no |
| A/duck/Cambodia/381W11M4/2013 | PA | 1608 | G | A | Pro530Pro | synonymous | 4.38% | This site falls into a region of PA that interacts with PB1 residues 1-25, and this interaction is critical for transcription. | no |
| A/Cambodia/W0112303/2012 | PA | 1691 | A | G | Gln556Gln | synonymous | 3.60% | This site falls into a region of PA that interacts with PB1 residues 1-25, and this interaction is critical for transcription. | no |
| A/Cambodia/X0128304/2013 | PA | 1727 | A | G | Asn568Ser | nonsynonymous | 2.40% | This site falls into a region of PA that interacts with PB1 residues 1-25, and this interaction is critical for transcription. | no |
| A/Cambodia/X0125302/2013 | PA | 1728 | G | A | Thr570Thr | synonymous | 2.03% | This site falls into a region of PA that interacts with PB1 residues 1-25, and this interaction is critical for transcription. | no |
| A/Cambodia/W0112303/2012 | PA | 1789 | T | C | Leu589Pro | nonsynonymous | 2.07% | This site falls into a region of PA that interacts with PB1 residues 1-25, and this interaction is critical for transcription. | no |
| A/Cambodia/X0207301/2013 | PA | 1841 | A | G | Glu610Gly | nonsynonymous | 2.15% | This site falls into a region of PA that interacts with PB1 residues 1-25, and this interaction is critical for transcription. | no |
| A/Cambodia/X0128304/2013 | PA | 1868 | A | G | Lys615Arg | nonsynonymous | 2.47% | An Asn at PA 615 has been associated with adaptation of avian influenza polymerases to humans. | yes |
| A/Cambodia/X0128304/2013 | PA | 1893 | A | G | Glu623Glu | synonymous | 2.52% | This site falls into a region of PA that interacts with PB1 residues 1-25, and this interaction is critical for transcription. | no |
| A/Cambodia/X0207301/2013 | PA | 1902 | A | G | Glu630Glu | synonymous | 3.07% | This site falls into a region of PA that interacts with PB1 residues 1-25, and this interaction is critical for transcription. | no |
| A/Cambodia/X0207301/2013 | PA | 1903 | A | G | Ser631Gly | nonsynonymous | 1.79% | A Ser at 631 enhances virulence of H5N1 in mice. | yes |
| A/Cambodia/X0128304/2013 | PA | 1999 | T | C | Ser659Pro | nonsynonymous | 6.79% | This site falls into a region of PA that interacts with PB1 residues 1-25, and this interaction is critical for transcription. | no |
| A/Cambodia/X1030304/2013 | PA | 2055 | A | G | Gly679Gly | synonymous | 6.98% | This site falls into a region of PA that interacts with PB1 residues 1-25, and this interaction is critical for transcription. | no |
| A/Cambodia/X0128304/2013 | HA | 149 | C | T | Thr41Ile | Nonsynonymous | 5.19% | No known function. | no |
| A/Cambodia/X0128304/2013 | HA | 163 | C | T | Gln46Stop | stop gained | 20.24% | No known function. | no |
| A/Cambodia/X0128304/2013 | HA | 299 | A | G | Glu91Gly | nonsynonymous | 6.33% | A Lys at 91 enhances α-2,6 binding. | yes |
| A/Cambodia/X1030304/2013 | HA | 306 | C | T | Val102Val | synonymous | 1.79% | No known function. | no |
| A/duck/Cambodia/083D1/2011 | HA | 394 | A | G | Lys129Glu | nonsynonymous | 9.63% | No known function. | no |
| A/Cambodia/V0401301/2011 | HA | 422 | A | G | His141Arg | nonsynonymous | 7.56% | No known function. | no |
| A/Cambodia/V0417301/2011 | HA | 422 | A | G | His141Arg | nonsynonymous | 17.50% | No known function. | no |
| A/Cambodia/V0417301/2011 | HA | 425 | A | G | Glu142Gly | nonsynonymous | 3.20% | Putative glycosylation site. | yes |
| A/Cambodia/V0401301/2011 | HA | 449 | C | T | Ala150Val | nonsynonymous | 20.24% | A Val at 150 confers enhanced α-2,6 sialic acid binding in H5N1 viruses. | yes |
| A/Cambodia/X0125302/2013 | HA | 449 | C | T | Ala150Val | nonsynonymous | 15.09% | A Val at 150 confers enhanced α-2,6 sialic acid binding in H5N1 viruses. | yes |
| A/Cambodia/X0128304/2013 | HA | 450 | T | C | His141His | synonymous | 5.69% | No known function | no |
| A/Cambodia/V0401301/2011 | HA | 497 | T | C | Leu166Pro | nonsynonymous | 4.28% | No known function | no |
| A/Cambodia/V0401301/2011 | HA | 513 | T | C | Ser171Ser | synonymous | 3.30% | Part of putative glycosylation motif that improves α-2,6 binding. | yes |
| A/Cambodia/V0401301/2011 | HA | 517 | T | C | Tyr173His | nonsynonymous | 5.04% | Residue involved in sialic acid recognition. | yes |
| A/Cambodia/V0401301/2011 | HA | 527 | T | C | Ile176Thr | nonsynonymous | 4.26% | No known function | no |
| A/Cambodia/X0128304/2013 | HA | 542 | A | C | Lys172Thr | nonsynonymous | 11.50% | Part of putative glycosylation motif that improves α-2,6 binding. | yes |
| A/Cambodia/V0401301/2011 | HA | 590 | C | T | Pro197Leu | nonsynonymous | 2.40% | No known function | no |
| A/Cambodia/V0401301/2011 | HA | 593 | A | G | Asn198Ser | nonsynonymous | 3.32% | A Lys at 198 confers α-2,6 sialic acid binding. | yes |
| A/Cambodia/V0401301/2011 | HA | 628 | C | A | Pro210Thr | nonsynonymous | 2.42% | No known function | no |
| A/Cambodia/V0417301/2011 | HA | 664 | A | G | Asn222Asp | nonsynonymous | 3.18% | No known function | no |
| A/Cambodia/V0401301/2011 | HA | 694 | A | G | Arg232Gly | nonsynonymous | 4.83% | No known function | no |
| A/Cambodia/V0401301/2011 | HA | 695 | G | A | Arg232Lys | nonsynonymous | 4.07% | No known function | no |
| A/Cambodia/X0128304/2013 | HA | 703 | A | G | Thr226Ala | nonsynonymous | 28.91% | An Ile at 226 enhanced α-2,6 sialic acid binding | yes |
| A/Cambodia/V0401301/2011 | HA | 713 | A | T | Gln238Leu | nonsynonymous | 2.80% | A Leu at 238 confers a switch from α-2,3 to α-2,6 sialic acid binding and is a determinant of mammalian transmission. | yes |
| A/Cambodia/V0417301/2011 | HA | 713 | A | T | Gln238Leu | nonsynonymous | 8.45% | A Leu at 238 confers a switch from α-2,3 to α-2,6 sialic acid binding and is a determinant of mammalian transmission. | yes |
| A/Cambodia/X0125302/2013 | HA | 713 | A | G | Gln238Arg | nonsynonymous | 40.30% | A Leu at 238 confers a switch from α-2,3 to α-2,6 sialic acid binding and is a determinant of mammalian transmission. | yes |
| A/Cambodia/V0401301/2011 | HA | 754 | A | G | Asn252Asp | nonsynonymous | 5.08% | No known function | no |
| A/duck/Cambodia/381W11M4/2013 | HA | 793 | G | A | Ala265Thr | nonsynonymous | 3.28% | No known function | no |
| A/Cambodia/W0112303/2012 | HA | 806 | T | C | Ala265Ala | synonymous | 5.62% | No known function | no |
| A/Cambodia/X0128304/2013 | HA | 811 | A | G | Asn262Asp | nonsynonymous | 3.38% | No known function | no |
| A/Cambodia/X0207301/2013 | HA | 919 | T | C | Phe307Leu | nonsynonymous | 19.82% | No known function | no |
| A/Cambodia/X1030304/2013 | HA | 919 | T | C | Phe307Leu | nonsynonymous | 6.65% | No known function | no |
| A/Cambodia/X0128304/2013 | HA | 992 | T | C | Val322Ala | nonsynonymous | 9.30% | No known function | no |
| A/Cambodia/X0207301/2013 | HA | 1056 | A | C | Ile352Ile | synonymous | 9.42% | No known function | no |
| A/Cambodia/V0417301/2011 | HA | 1071 | A | G | Glu357Glu | synonymous | 6.35% | No known function | no |
| A/Cambodia/X0207301/2013 | HA | 1071 | A | G | Glu357Glu | synonymous | 5.83% | No known function | no |
| A/duck/Cambodia/Y0224304/2014 | HA | 1103 | G | A | Val363Ile | nonsynonymous | 6.32% | No known function | no |
| A/duck/Cambodia/Y0224301/2014 | HA | 1131 | A | T | Gly377Gly | synonymous | 3% | No known function | no |
| A/Cambodia/X0219301/2013 | HA | 1221 | C | T | Thr407Thr | synonymous | 17.86% | No known function | no |
| A/Cambodia/X0128304/2013 | HA | 1281 | C | A | Asn418Lys | nonsynonymous | 27.90% | No known function | no |
| A/duck/Cambodia/Y0224304/2014 | HA | 1360 | A | G | Glu448Glu | synonymous | 2.37% | No known function | no |
| A/Cambodia/X0128304/2013 | HA | 1410 | A | G | Val461Val | synonymous | 10.74% | No known function | no |
| A/Cambodia/V0417301/2011 | HA | 1506 | A | G | Thr502Thr | synonymous | 15.22% | No known function | no |
| A/Cambodia/X0128304/2013 | HA | 1535 | A | G | Tyr503Cys | nonsynonymous | 4.98% | No known function | no |
| A/Cambodia/X0128304/2013 | HA | 1629 | A | G | Ser534Ser | synonymous | 10.14% | No known function | no |
| A/Cambodia/V0417301/2011 | NP | 146 | G | A | Gly37Gly | synonymous | 3.90% | RNA-binding domain | no |
| A/duck/Cambodia/Y0224304/2014 | NP | 207 | C | T | Asn59Asn | synonymous | 2.70% | RNA-binding domain | no |
| A/Cambodia/V0401301/2011 | NP | 293 | G | A | Arg98Gln | nonsynonymous | 2.42% | RNA-binding domain | no |
| A/Cambodia/V0401301/2011 | NP | 342 | G | A | Glu114Glu | synonymous | 1.96% | RNA-binding domain | no |
| A/Cambodia/W0112303/2012 | NP | 356 | T | C | Leu108Pro | nonsynonymous | 2.23% | RNA-binding domain | no |
| A/Cambodia/W0112303/2012 | NP | 358 | A | G | Ile109Val | nonsynonymous | 3.20% | RNA-binding domain | no |
| A/duck/Cambodia/Y0224304/2014 | NP | 378 | C | T | Ile116Ile | synonymous | 20.43% | RNA-binding domain | no |
| A/duck/Cambodia/381W11M4/2013 | NP | 384 | A | G | Gln117Arg | nonsynonymous | 3.53% | RNA-binding domain | no |
| A/duck/Cambodia/Y0224304/2014 | NP | 402 | C | T | Asn124Asn | synonymous | 2.87% | RNA-binding domain | no |
| A/Cambodia/V0401301/2011 | NP | 496 | C | G | Leu166Val | nonsynonymous | 3.77% | RNA-binding domain | no |
| A/duck/Cambodia/Y0224304/2014 | NP | 539 | C | T | Ser170Leu | nonsynonymous | 6.13% | RNA-binding domain | no |
| A/Cambodia/X0128304/2013 | NP | 542 | A | G | Gln168Gln | synonymous | 1.92% | RNA-binding domain | no |
| A/duck/Cambodia/Y0224304/2014 | NP | 593 | C | T | Thr188Ile | nonsynonymous | 4.76% | No known function | no |
| A/Cambodia/V0401301/2011 | NP | 603 | C | A | Val201Val | synonymous | 2.81% | Nuclear targeting motif | yes |
| A/duck/Cambodia/Y0224304/2014 | NP | 633 | C | T | Ile201Ile | synonymous | 9.23% | Nuclear targeting motif | yes |
| A/duck/Cambodia/Y0224304/2014 | NP | 636 | C | T | Asn202Asn | synonymous | 3.70% | Nuclear targeting motif | yes |
| A/Cambodia/V0401301/2011 | NP | 659 | A | G | Glu220Gly | nonsynonymous | 2.17% | Region involved in NP-NP association | no |
| A/duck/Cambodia/Y0224304/2014 | NP | 674 | C | T | Thr215Ile | nonsynonymous | 3.69% | Nuclear targeting motif | yes |
| A/Cambodia/X0128304/2013 | NP | 712 | T | C | Ile225Thr | nonsynonymous | 1.82% | Region involved in NP-NP association | no |
| A/Cambodia/X0128304/2013 | NP | 770 | G | A | Glu244Glu | synonymous | 15.70% | Region involved in NP-NP association | no |
| A/Cambodia/X0128304/2013 | NP | 771 | A | G | Ser245Gly | nonsynonymous | 2.04% | Region involved in NP-NP association | no |
| A/Cambodia/X0128304/2013 | NP | 774 | A | G | Arg246Gly | nonsynonymous | 2.24% | Region involved in NP-NP association | no |
| A/Cambodia/V0401301/2011 | NP | 799 | A | G | Arg267Gly | nonsynonymous | 3.36% | Region involved in NP-NP association | no |
| A/Cambodia/W0112303/2012 | NP | 804 | T | A | Ile257Ile | synonymous | 1.92% | Region involved in NP-NP association | no |
| A/Cambodia/X0219301/2013 | NP | 814 | C | T | Ala260Val | nonsynonymous | 2.02% | Region involved in NP-NP association | no |
| A/Cambodia/V0417301/2011 | NP | 1017 | T | C | Leu328Leu | synonymous | 6.72% | Region involved in NP-NP association | no |
| A/Cambodia/X0128304/2013 | NP | 1055 | G | A | Glu339Glu | synonymous | 14.89% | Region involved in NP-NP association | no |
| A/Cambodia/X0219301/2013 | NP | 1184 | T | C | Ser383Ser | synonymous | 6.30% | Region involved in NP-NP association | no |
| A/Cambodia/X0207301/2013 | NP | 1217 | G | A | Arg391Arg | synonymous | 3.81% | Region involved in NP-NP association | no |
| A/duck/Cambodia/083D1/2011 | NP | 1241 | C | T | Ala403Val | nonsynonymous | 2.92% | Region involved in NP-NP association | no |
| A/duck/Cambodia/083D1/2011 | NP | 1242 | A | T | Ala403Ala | synonymous | 2.52% | Region involved in NP-NP association | no |
| A/Cambodia/X0128304/2013 | NP | 1319 | A | G | Ala427Ala | synonymous | 6.31% | Region involved in NP-NP association | no |
| A/Cambodia/V0417301/2011 | NA | 146 | G | A | Glu47Lys | nonsynonymous | 8.89% | A deletion in this part of NA has been linked to host range. | yes |
| A/duck/Cambodia/083D1/2011 | NA | 181 | A | G | Lys58Glu | nonsynonymous | 17.89% | A deletion in this part of NA has been linked to host range. | yes |
| A/Cambodia/V0417301/2011 | NA | 280 | A | G | Lys91Lys | synonymous | 3.70% | No known function | no |
| A/duck/Cambodia/083D1/2011 | NA | 297 | C | A | Val96Val | synonymous | 17.95% | No known function | no |
| A/Cambodia/X0207301/2013 | NA | 323 | C | A | His106Gln | nonsynonymous | 2.83% | No known function | no |
| A/Cambodia/V0401301/2011 | NA | 462 | C | G | Pro149Arg | nonsynonymous | 1.92% | An Ala at 149 is responsible for reduced zanamivir sensitivity in an H5N1 virus. 149 is part of the NA catalytic site. | no |
| A/Cambodia/X0128304/2013 | NA | 497 | C | T | Cys164Cys | synonymous | 8.01% | No known function | no |
| A/Cambodia/X0128304/2013 | NA | 527 | A | G | Gly174Gly | synonymous | 2.95% | No known function | no |
| A/Cambodia/V0401301/2011 | NA | 553 | C | T | Asp179Asp | synonymous | 1.73% | Oseltamivir binding site | no |
| A/Cambodia/V0401301/2011 | NA | 571 | A | G | Val185Val | synonymous | 2.49% | No known function | no |
| A/Cambodia/V0401301/2011 | NA | 656 | G | A | Val214Ile | nonsynonymous | 3.98% | No known function | no |
| A/Cambodia/V0417301/2011 | NA | 658 | T | C | Tyr217Tyr | synonymous | 34.25% | No known function | no |
| A/Cambodia/X0125302/2013 | NA | 722 | A | G | Glu239Glu | synonymous | 1.82% | No known function | no |
| A/duck/Cambodia/Y0224301/2014 | NA | 733 | A | G | Lys242Lys | synonymous | 2.65% | No known function | no |
| A/Cambodia/V0401301/2011 | NA | 824 | T | A | Cys270Ser | nonsynonymous | 3.01% | No known function | no |
| A/Cambodia/X0207301/2013 | NA | 881 | T | C | Tyr292Tyr | synonymous | 3.15% | No known function | no |
| A/Cambodia/V0401301/2011 | NA | 985 | A | G | Ala323Ala | synonymous | 3.80% | No known function | no |
| A/Cambodia/V0401301/2011 | NA | 1046 | A | G | Ser344Gly | nonsynonymous | 4.56% | Oseltamivir binding site | no |
| A/Cambodia/V0401301/2011 | NA | 1047 | G | A | Ser344Asn | nonsynonymous | 2.11% | Oseltamivir binding site | no |
| A/Cambodia/X0128304/2013 | NA | 1062 | A | G | Met353Val | nonsynonymous | 4.51% | No known function | no |
| A/Cambodia/V0401301/2011 | NA | 1108 | C | T | Asp364Asp | synonymous | 2.42% | No known function | no |
| A/Cambodia/V0401301/2011 | NA | 1138 | A | G | Val374Val | synonymous | 3.09% | No known function | no |
| A/Cambodia/X0128304/2013 | NA | 1257 | A | G | Thr418Ala | nonsynonymous | 2.52% | No known function | no |
| A/Cambodia/X0125302/2013 | NA | 1304 | G | A | Val433Val | synonymous | 4.63% | No known function | no |
| A/Cambodia/X0207301/2013 | M1 | 100 | G | A | Lys27Lys | synonymous | 29.01% | Membrane-binding region | no |
| A/Cambodia/X0125302/2013 | M1 | 101 | G | A | Asp30Asn | nonsynonymous | 3.93% | Membrane-binding region | no |
| A/Cambodia/X0128304/2013 | M1 | 102 | A | G | Gln26Arg | nonsynonymous | 8.57% | Membrane-binding region | no |
| A/Cambodia/X0128304/2013 | M1 | 158 | T | G | Trp45Gly | nonsynonymous | 5.10% | Membrane-binding region | no |
| A/Cambodia/X0207301/2013 | M1 | 496 | T | C | His159His | synonymous | 1.96% | Membrane-binding region | no |
| A/Cambodia/X0128304/2013 | M1 | 528 | T | C | Ile168Thr | nonsynonymous | 2.61% | vRNP binding region | no |
| A/Cambodia/X0128304/2013 | M1 | 557 | A | G | Arg178Gly | nonsynonymous | 1.80% | vRNP binding region | no |
| A/Cambodia/V0401301/2011 | M1 | 569 | G | A | Gly185Asp | nonsynonymous | 2.39% | vRNP binding region | no |
| A/duck/Cambodia/083D1/2011 | M1 | 580 | G | T | Gly194Stop | stop gained | 2.70% | vRNP binding region | no |
| A/duck/Cambodia/083D1/2011 | M1 | 596 | C | T | Ala199Val | nonsynonymous | 2.92% | vRNP binding region | no |
| A/Cambodia/X0128304/2013 | M1 | 619 | G | A | Gln198Gln | synonymous | 5.85% | vRNP binding region | no |
| A/duck/Cambodia/PV027D1/2010 | M1 | 621 | C | T | Ala199Val | nonsynonymous | 4.56% | vRNP binding region | no |
| A/Cambodia/X0128304/2013 | M1 | 638 | A | G | Ile205Val | nonsynonymous | 2.67% | vRNP binding region | no |
| A/Cambodia/X0207301/2013 | M1 | 679 | G | A | Gly220Gly | synonymous | 6.28% | vRNP binding region | no |
| A/Cambodia/X1030304/2013 | M1 | 703 | G | A | Ala227Thr | nonsynonymous | 1.88% | vRNP binding region | no |
| A/Cambodia/X1030304/2013 | M1 | 717 | T | C | Asp231Asp | synonymous | 3.05% | vRNP binding region | no |
| A/Cambodia/X0128304/2013 | M1 | 742 | C | G | Ala239Ala | synonymous | 5.10% | vRNP binding region | no |
| A/Cambodia/X1030304/2013 | M1 | 751 | A | C | Arg243Arg | synonymous | 2.61% | vRNP binding region | no |
| A/Cambodia/X0128304/2013 | M2 | 742 | C | G | Pro10Arg | nonsynonymous | 5.10% | No known function | no |
| A/Cambodia/X1030304/2013 | M2 | 751 | A | C | Lys13Asn | nonsynonymous | 2.61% | No known function | no |
| A/Cambodia/X1030304/2013 | M2 | 861 | G | A | Cys50Tyr | nonsynonymous | 2.03% | A Cys at position 50 is a palmitoylation site that enhances virulence. | yes |
| A/Cambodia/X0125302/2013 | M2 | 882 | A | G | Arg61Gly | nonsynonymous | 2.28% | No known function | no |
| A/Cambodia/V0417301/2011 | M2 | 955 | A | C | Asp85Ala | nonsynonymous | 4.07% | No known function | no |
| A/Cambodia/V0401301/2011 | M2 | 978 | G | A | Trp89Stop | stop gained | 6.87% | No known function | no |
| A/Cambodia/X0125302/2013 | NS1 | 298 | A | G | Glu92Glu | synonymous | 2.49% | An Asp at 92 helps to confer cytokine resistance in H5N1 viruses. | yes |
| A/Cambodia/X0128304/2013 | NS1 | 302 | A | G | Glu92Glu | synonymous | 29.06% | An Asp at 92 helps to confer cytokine resistance in H5N1 viruses. | yes |
| A/Cambodia/X1030304/2013 | NS1 | 379 | A | G | Lys121Lys | synonymous | 1.93% | Part of NS1 effector domain, which interacts with many host proteins | yes |
| A/Cambodia/X0128304/2013 | NS1 | 413 | C | T | Phe129Phe | synonymous | 1.77% | Part of NS1 effector domain, which interacts with many host proteins | yes |
| A/Cambodia/X0128304/2013 | NS1 | 502 | C | T | Pro159Leu | nonsynonymous | 2.88% | Part of the NS1 nuclear export signal mask. | yes |
| A/Cambodia/X0128304/2013 | NS1 | 554 | C | T | Leu176Leu | synonymous | 3.06% | Part of NS1 effector domain, which interacts with many host proteins | yes |
| A/Cambodia/X0207301/2013 | NS1 | 609 | A | G | Arg199Arg | synonymous | 4.59% | Part of NS1 effector domain, which interacts with many host proteins | yes |
| A/duck/Cambodia/Y0224301/2014 | NS1 | 646 | T | C | Leu207Pro | nonsynonymous | 2.22% | NS1 flexible tail, which interacts with host machinery. | yes |
| A/duck/Cambodia/Y0224301/2014 | NS1 | 654 | C | T | Pro210Ser | nonsynonymous | 2.55% | NS1 flexible tail, which interacts with host machinery. | yes |
| A/Cambodia/X0128304/2013 | NEP | 554 | C | T | Ser24Leu | nonsynonymous | 3.06% | No known function. | no |
| A/Cambodia/X0207301/2013 | NEP | 609 | A | G | Glu47Gly | nonsynonymous | 4.59% | This site was implicated in enhanced virulence of H5N1 in ferrets. | yes |
| A/duck/Cambodia/Y0224301/2014 | NEP | 646 | T | C | Phe55Leu | nonsynonymous | 2.22% | No known function | no |
| A/duck/Cambodia/Y0224301/2014 | NEP | 654 | C | T | Ser57Ser | synonymous | 2.55% | No know function | no |
